# Thrombospondin 1 Mediates Autophagy Upon Inhibition of the Rho-Associated Protein Kinase Inhibitor

**DOI:** 10.3390/cells13221907

**Published:** 2024-11-18

**Authors:** Kirk Patrick Carreon Catral, Choi-Yee Tse, Wei-Ying Yang, Choi-Ying Ling, Oi-Lam Kwok, Kit-Ying Choy, Da-Qian Lu, Jing-Fang Bian, Thomas Chuen Lam, Dennis Yan-Yin Tse, Samantha Sze-Wan Shan

**Affiliations:** 1 School of Optometry, The Hong Kong Polytechnic University, Hong Kong, China; kpatrick.catral@connect.polyu.hk (K.P.C.C.); choi-yee.tse@connect.polyu.hk (C.-Y.T.); alvia.yang@polyu.edu.hk (W.-Y.Y.); challie.ling@polyu.edu.hk (C.-Y.L.); oi-lam.kwok@connect.polyu.hk (O.-L.K.); kitying.choy@polyu.edu.hk (K.-Y.C.); da-qian.lu@connect.polyu.hk (D.-Q.L.); jingfang.jf.bian@polyu.edu.hk (J.-F.B.); thomas.c.lam@polyu.edu.hk (T.C.L.); 2 Centre for Eye and Vision Research (CEVR), 17W Hong Kong Science Park, Hong Kong, China; 3 Research Centre for SHARP Vision (RCSV), The Hong Kong Polytechnic University, Hong Kong, China; 4 Research Centre for Chinese Medicine (RCMI), The Hong Kong Polytechnic University, Hong Kong, China

**Keywords:** autophagy, Thrombospondin-1, ROCK inhibitor, AMD, signaling pathway

## Abstract

Age-related macular degeneration (AMD) is a degenerative eye disease leading to central vision loss and is characterized by dysregulated autophagy of the retinal pigment epithelium (RPE) layer. Recent studies have suggested that rho-associated protein kinase (ROCK) inhibitors may enhance autophagy in neurodegenerative diseases and promote the survival of RPE cells. This study investigated the effect of ROCK inhibitors on autophagy gene expression and autophagic vacuole formation in a human RPE (ARPE-19) cell line. The highly selective and potent ROCK inhibitor Y-39983 enhanced the expression of autophagy genes in ARPE-19 cells and increased autophagic vacuole formation. A proteomic analysis using mass spectrometry was performed to further characterize the effects of ROCK inhibition at the protein level. Y-39983 downregulated thrombospondin-1 (THBS1), and suppression of THBS1 in ARPE-19 cells resulted in an increase in autophagic vacuole formation. Our data showed that ROCK inhibitor-induced autophagy was mediated by THBS1 downregulation. We identified ROCK and THBS1 as potential novel therapeutic targets in AMD.

## 1. Introduction

Age-related macular degeneration (AMD) is one of the leading causes of visual impairment and blindness. A systemic analysis of the causes of blindness worldwide conducted by the Vision Loss Expert Group showed that AMD was responsible for approximately 4.9% and 6.6% of global blindness (defined as visual acuity below 3/60 in the better eye) in 1990 and 2010, respectively [1]. Wong, Su [2] estimated that the projected number of people in the age range of 45–85 with AMD will be 288 million in 2040. The global prevalence of AMD is expected to increase mainly due to the aging global population. AMD management varies based on the different stages. AMD can be classified into four categories according to the size and number of drusen, presence of pigmentary change, development of geographic atrophy, and evidence of neovascularization [3,4].

The retinal pigmented epithelium (RPE) is responsible for the phagocytosis and subsequent autophagic degradation of shed photoreceptor outer segments (POSs) [5]. Reduced autophagic flux in RPE results in incomplete autophagic degradation of phagocytosed POS and damaged intracellular organelles, leading to the accumulation of drusen, which are rich in lipofuscin [6]. Autophagy is believed to play an important role in the pathogenesis of AMD [7,8]. Autophagy activity decreases with age, causing an imbalance between the protein damage rate and turnover rate, resulting in the accumulation of altered proteins and organelles [9]. Impaired autophagy is more detrimental in long-lived post-mitotic cells, such as neurons and cardiac myocytes, compared to short-lived post-mitotic cells, such as peripheral blood, and intestinal epithelial cells [9]. While short-lived post-mitotic cells can renew through the proliferation and differentiation of stem cells to replace the proportion of damaged biological structures, long-lived post-mitotic cells are more susceptible to the chronic accumulation of waste products because of a very low or even zero replacement rate. Autophagic activity declines with increasing age in organs largely composed of long-lived post-mitotic cells, including the liver, heart, and retina. Accordingly, these organs tend to be more susceptible to conditions resulting from the accumulation of waste materials during aging [10,11,12].

Activation of Rho-associated protein kinase (ROCK) suppresses autophagy, and ROCK inhibitors are capable of activating autophagy in multiple organs and tissues, including the kidney, heart, brain, and cornea [13,14,15,16]. ROCK inhibitors have shown positive effects in an animal model of Huntington’s disease, reduced oligomeric tau protein in cell culture and improved parkin-mediated mitophagy in vitro [16,17], suggesting that improved autophagy may facilitate the clearance of pathologic protein accumulation in neurodegenerative diseases. Consistently, impaired autophagy has been associated with the accumulation of alpha-synuclein [18], polyglutamine [19], and tau and amyloid-beta [18]. Consequently, AMD, a neurodegenerative disease characterized by the accumulation of lipofuscin, may benefit from the upregulation of autophagy through ROCK inhibition. Studies have shown that ROCK inhibitors may be beneficial to RPE cell survival and have therapeutic potential for AMD. Hollanders, Van Bergen [20] evaluated the therapeutic benefit of ROCK inhibitor AMA0428 in an animal model of neovascular AMD. Processes involved in neovascular AMD (inflammation, angiogenesis, vascular leakage, and fibrosis) were found to be reduced by the administration of AMA0428. A study conducted by Ni, Qin [21] found that ROCK inhibitor Y-27632 significantly increased ARPE-19 cell proliferation and protected the cells from apoptosis. While showing that ROCK inhibition benefited the survival of RPE cells and improved AMD pathology, neither study assessed autophagy. Therefore, the effect of ROCK inhibitors on retinal autophagy and the relevance of such effects on AMD pathology remain unclear. To the best of our knowledge, no study has investigated the effects of ROCK inhibitors on autophagy and survival in RPE cells. Therefore, this study quantified autophagy gene expression and autophagic vacuoles following treatment of ARPE-19 cells with the highly selective and potent ROCK inhibitor Y-39983 [22,23]. Y-39983 is a novel ROCK inhibitor derived from Y-27632, the first ROCK inhibitor [23]. Its inhibitory effect on ROCK is 30 times stronger than that of Y-27632 and has a higher stability [23]. The higher potency of Y-39983 is beneficial, as a lower concentration can exert the desired magnitude of effect, thus lowering the chances of toxicity. In addition, the effects of Y-39983 on protein abundance were comprehensively evaluated using a proteomic analysis with a novel ZenoTOF SWATH-based LC-MS/MS approach, which offers high MS/MS sensitivity for protein identification and quantification [24]. To our knowledge, this is the first effort to quantify differential protein expression using a Zeno SWATH DIA protocol, followed by an online OneOmics platform analysis on ROCK inhibitor-treated ARPE-19 cells. The results facilitate the identification of molecular candidates for novel anti-AMD drug development.

## 2. Materials and Methods

### 2.1. Human Retinal Pigmented Epithelium (ARPE-19) Cell Culture

Human ARPE-19 cells (ATCC, Manassas, VA, USA) were cultured in Dulbecco’s Modified Eagle’s Medium (DMEM)/F12 (Invitrogen, Waltham, MA, USA), which contains 10% fetal bovine serum (FBS; Gibco, Waltham, MA, USA) and 1% penicillin–streptomycin (PS; Gibco, Waltham, MA, USA). Cells were incubated at 37 °C in a humidified atmosphere of 5% carbon dioxide (CO_2_). The medium was replaced every 3 days until they were 90% confluent. The cells were then subcultured into new 75 cm^2^ flasks.

### 2.2. Cell Viability

Cell viability was assessed by the trypan blue dye exclusion test. ARPE-19 cells were plated in 6-well plates at a seeding density of 1.0 × 10^5^ cells/mL with a serum-free medium overnight at 37 °C and 5% CO_2_. After incubating the cells with different concentrations of Y-39983 (vehicle, 0.01 µM, 0.1 µM, and 1 µM) for 48 h, cells were detached by 0.25% trypsin and transferred to reaction tubes, and 0.4% trypan blue solution was added to each tube (1:1; 0.4% trypan blue:suspended cells), gently mixed, and left at room temperature for at least 5 min. From each tube, 10 µL was transferred into a hemocytometer for quantification of the total and viable cells to determine the percentage of cell viability.

### 2.3. Cell Morphology

ARPE-19 cells were plated in 6-well plates at a seeding density of 1.5 × 10^5^ cells/mL with serum-free medium overnight at 37 °C and 5% CO_2_. To study the effects of the ROCK inhibitor on the cell morphology of ARPE-19 cells, different concentrations (vehicle, 0.01 µM, 0.1 µM, and 1 µM) of Y-39983 (MedChem Express, Princeton, NJ, USA) were added, and cells were incubated for 48 h prior to taking photographs under a Zeiss Primovert inverted microscope using Labscope software (V.3.4., Zeiss, Germany).

### 2.4. Real-Time-Quantitative Polymerase Chain Reaction (RT-qPCR)

Total RNA of ARPE-19 cells was extracted using the Qiagen RNeasy Micro Kit (Qiagen, Hilden, Germany) and quantified. Reverse transcription of RNA to complementary DNA (cDNA) was performed using the High-Capacity cDNA Reverse Transcription Kit (Thermo Fisher Scientific, Waltham, MA, USA), followed by qPCR using the LightCycler 480 SYBR Green I Master mix (Roche Applied Science, Mannheim, Germany) with primers specific for the target genes: *ATG5*, *ATG7*, *Nrf2*, *p62*, *TFEB*, and *THBS1* and the internal reference gene *GAPDH* (Table 1). The qPCR reaction was performed in 96-well plates on the ROCHE LightCycler 480 (Roche Diagnostics Ltd., Basal, Switzerland). In each 10 μL reaction, 5 μL of LightCycler 180 SYBR Green I Master Mix, 1.5 μL of PCR-graded water, 1.5 μL of cDNA template, and 1 μL of 10 μM primers (for both forward and reverse primers) were used. The thermal cycling conditions were 95 °C for 5 min, followed by 45 amplification cycles (95 °C for 30 s, 61 °C for 30 s, and 72 °C for 30 s). A melting curve analysis was performed to rule out primer–dimer formation or non-specific product amplification. A negative control sample without a template was included on each plate. Data were analyzed using LightC480 software (V 2.9.) [25].

### 2.5. Protein Extraction and Digestion for MS

Cell pellets were lysed with 50 µL of lysis buffer (5% SDS and 50 mM TEAB, pH 7.55) in a 2 mL tissue homogenizing CKMix standard tube (Precellys Lysing Kit, Bertin Corp, Rockville, MD, USA). The homogenization process was done in a liquid nitrogen cooling chamber at 5800 rpm for 30 s at 4 °C. Cell lysates were centrifuged, and the supernatant was collected. The protein concentration was measured using the Pierce™ Rapid Gold BCA Protein Assay Kit (Thermo Fisher Scientific, Waltham, MA, USA) according to the user manual.

Protein digestion was done using the S-trap micro-spin column protocol (ProtiFi, Fairport, NY, USA) due to its effectiveness for cell lysate samples [26,27]. In brief, 50 µg of protein samples were reduced with tris(2-carboxyethyl) phosphine hydrochloride at a final concentration of 5 mM and alkylated with methyl methanethiosulfonate to a final concentration of 20 mM. Phosphoric acid was added to ensure denaturation of the proteins and efficient trapping. S-trap binding buffer was added next, and samples were placed in a micro-spin column for centrifugation to trap the proteins. Samples were subsequently washed with the S-trap protein binding buffer. Trypsin (1:10, *w*/*w*) was then added and incubated for 1 h at 47 °C for digestion. Samples were then sequentially eluted with 50 mM TEAB, 0.2% formic acid, and 50% acetonitrile. These eluents were pooled together and dried in a Refrigerated CentriVap Vacuum Concentrator (Labconco Corporation, Kansas City, MO, USA) at 4 °C. Peptides were resuspended with 0.1% formic acid, and the peptide concentration was determined using the Pierce™ Quantitative Peptide Assay (Thermo Fisher Scientific, Waltham, MA, USA).

### 2.6. Zeno SWATH Acquisition and Bioinformatic Analysis

With a 1 µg peptide injection amount, each biological sample (n = 3 in each group) was loaded with 2 technical replicates into a microflow liquid chromatography (LC) system. Specifically, peptides of each sample were loaded into a nanoEase™ M/Z HSS T3 column (100Å, 1.8 µm, 300 × 150 mm; Waters Corporation, Milford, MA, USA) with a 30-min LC gradient elution at a flow rate of 10 µL/min, which was interfaced with a ZenoTOF™ 7600 mass spectrometer (SCIEX, Framingham, MA, USA) operating under the Zeno SWATH acquisition protocol with 64 variable isolation windows (400 *m*/*z* to 750 *m*/*z*).

The source and gas parameters of the ZenoTOF 7600 were set as follows: ion source gas 1:30 psi, ion source gas 2:30 psi, curtain gas: 35 psi, CAD gas: 7, temperature: 300 °C, and column temperature: 40 °C. DIA was in positive polarity using a 5500 V spray voltage. The Zeno SWATH acquisition parameters were set as follows: for the TOF MS, start mass: 400 Da, stop mass: 1250 Da, declustering potential: 80 V, DP spread: 0 V, collision energy: 10 V, CE spread: 0 V, and accumulation time: 0.1 s. For the TOF MSMS, Fragmentation mode: CID, start mass: 100 Da, stop mass: 1500 Da, accumulation time: 0.02 s, and dynamic collision energy: enable with a charge state of 2, Zeno pulsing.

For protein identification and quantification, the generated raw Zeno SWATH files (.wiff) were subjected to a DIA-NN 1.8.1 analysis [28], with the aid of an in silico-predicted spectral library generated from a UniProt (https://www.uniprot.org, accessed on 7 July 2023) FASTA database of the human proteome (UP000005640, accessed in July 2023) [29,30]. The resulting report file from DIA-NN was imported into the OneOmics software (SCIEX, Framingham, MA, USA, https://sciex.com/products/software/oneomics, accessed on 9 August 2023) via PeakView 2.1 (SCIEX, Framingham, MA, USA) for data quality control and statistical evaluation of the feature abundance. The mass spectrometry proteomics data were also deposited to the ProteomeXchange Consortium via the PRIDE partner repository with the dataset identifier PXD051479.

Within the OneOmics software, samples were grouped as control or Y-39983-treated biological replicates and their technical replicates. The resulting analyzed protein list from OneOmics was filtered with criteria that met the thresholds of 75% confidence, 0.15 reproducibility, and at least 2 matching peptides per protein. The cut-off values of DEPs were set to a fold change (FC) of ≥1.3 or ≤−1.3 (i.e., log2FC ≥ 0.38 or log2FC ≤ −0.38) and *p*-value < 0.05.

### 2.7. Pathway Analysis for Significant Regulated Proteins

Pathway analysis was conducted using iPathwayGuide software (Advaita Corporation, Ann Arbor, MI, USA, https://advaitabio.com/bioinformatics/ipathwayguide/, 9 August 2023). This tool utilizes an “impact analysis” approach, predicting the significance of proteins based on the roles, functions, positions, and interactions of the genes they encode [31,32]. All the significant DEPs (log2FC ≥ 0.38 or log2FC ≤ −0.38, *p*-value < 0.05.) found by OneOmics were imported into iPathwayGuide commercial software (https://advaitabio.com/bioinformatics/ipathwayguide/, accessed on 9 August 2023) for pathway analysis.

Significance was assessed using Fisher’s exact test for *p*-value calculation, with adjustments for multiple comparisons made via the Bonferroni correction. Pathways were considered significantly enriched when they exhibited adjusted *p*-values below 0.05.

### 2.8. Western Blot Analysis

The ARPE-19 cells were lysed in lysis buffer (7 M Urea, 2 M Thiourea, 30 mM TRIS, 1% ASB14, 2% CHAPS, and protease inhibitor cocktail, Roche Diagnostics Ltd., Basal, Switzerland) and sonicated for 1 h on ice, followed by centrifugation at 13,000× g for 20 min, and collection of the supernatants. Total protein concentration in the supernatants was quantified with the Bio-Rad Protein Assay (Bio-Rad Laboratories, Hercules, CA, USA). Total protein (25 µg) was mixed with the loading buffer (0.3 M Tris, 10% SDS, 50% *vol*/*vol* glycerol, 3.6 M beta-mercaptoethanol, and 0.5% bromophenol blue); heated at 95 °C for 5 min; separated in a 7% or 10% SDS-PAGE gel; and transferred into a PVDF membrane (Bio-Rad Laboratories, Hercules, CA, USA). The PVDF membrane was then incubated with the anti-THBS1 primary antibody (R&D systems, Minneapolis, MN, USA) at 4 °C overnight. After washing, the membrane was incubated with anti-goat IgG conjugated with horseradish peroxidase (Zymed Laboratories, San Francisco, CA, USA). Anti-GAPDH antibody (Calbiochem, San Diego, CA, USA) was used as a loading control. The protein expression was then visualized with the Pierce SuperSignal West Pico Chemiluminescent substrate (Thermo Fisher Scientific, Waltham, MA, USA).

### 2.9. Downregulation of THBS1 by siRNA

Gene silencing was performed using a *THBS1*-siRNA and a negative control siRNA. Both *THBS1*-specific (assay ID s14100) and negative control siRNA were purchased from Invitrogen (Thermo Fisher Scientific, Waltham, MA, USA). This non-silencing siRNA had no known homology to other mammalian genes, and 100 pmol siRNAs were transfected into ARPE-19 cells using Lipofectamine 3000 transfection reagent (Invitrogen, Waltham, MA, USA). After 24 h of incubation, the cells were processed for RT-qPCR.

### 2.10. Autophagy Assay

Autophagic vacuole formation is a measure of autophagy and was measured to investigate the relevance of THBS1 in autophagy in ARPE-19 cells in the presence of Y-39983 and THBS1 inhibitor peptides. The leucine-serine-lysine-leucine (LSKL) peptide (AnaSpec, Fermont, CA, USA) is a selective THBS1 inhibitor that inhibits THBS1-mediated TGF-β activation, while the serine-leucine-leucine-lysine (SLLK) peptide (AnaSpec) is a THBS1 inhibitor scramble peptide serving as a control. The cells were co-incubated with Y-39983 and either SLLK or LSKL peptide (5 μM) for 48 h. Autophagic vacuole formation was measured using an autophagy assay kit (Abcam, Cambridge, UK), according to the manufacturer’s instructions. The assay selectively labels autophagic vacuoles with a fluorescent dye, and the fluorescent intensity of the cells was quantified using a BD Accuri C6 Flow Cytometer (BD, Franklin Lakes, NJ, USA).

### 2.11. Statistical Analysis

Statistical analysis was performed with GraphPad Prism 10.1.2 (GraphPad Software Inc, San Diego, CA, USA). Student’s *t*-test or one-way analysis of variance (ANOVA) was used to compare the means between the control and treatment groups. A *p*-value < 0.05 was considered statistically significant. All data were represented as the mean ± standard error of the mean (SEM), and error bars were represented as SEM.

## 3. Results

### 3.1. Y-39983 Induced Changes in Cell Morphology at High Concentrations but Did Not Affect Cell Viability

**Figure 1:**
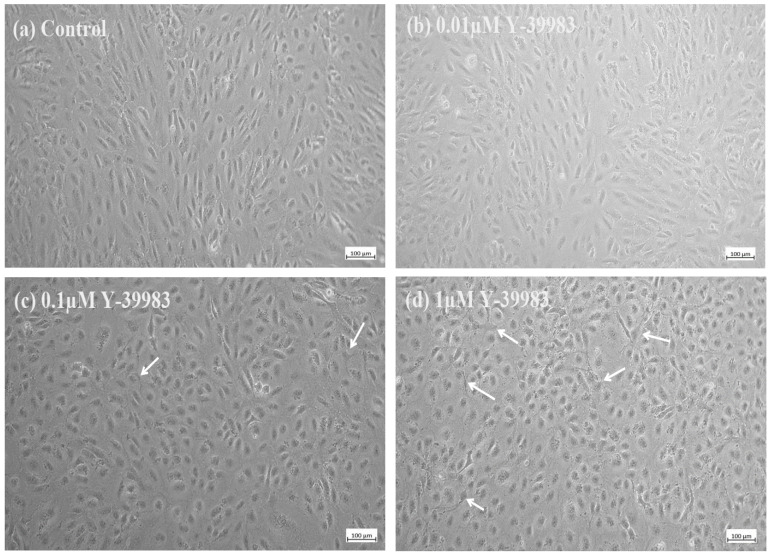
Typical phase-contrast microscopic images of ARPE-19 cells incubated with or without Y-39983. ARPE-19 cells incubated with (**a**) the control and (**b**–**d**) Y-39983 at different concentrations. White arrows show satellite-shaped morphology of ARPE-19 cells after treatment at higher concentrations of Y-39983 (n = 6). Scale bar = 100 µm.

To characterize the effect of Y-39983 on cell morphology, ARPE-19 cells were treated with Y-39983 (0.01–1 μM) or the vehicle control for 48 h before taking phase-contrast microscopic images. Vehicle-treated control cells and cells treated with a low Y-39938 concentration (0.01 µM) displayed a fibroblast-like appearance (Figure 1a,b) on phase-contrast microscopy. A satellite-shaped and cuboid appearance of cells was observed in ARPE-19 cells after treatment with 1 and 0.1 µM Y-39983 (Figure 1c,d).

The cell viability of ARPE-19 cells treated with various concentrations of Y-39983 was assessed. The cells were either treated with the vehicle or 0.01 µM, 0.1 µM, or 1 µM of Y-39983 for 48 h prior to measuring cell viability with the trypan blue assay. Y-39983 did not significantly affect the viability of ARPE-19 cells (Supplementary Figure S1).

### 3.2. Y-39983 Induced Autophagy Gene Expression and Autophagy Vacuole Assessment

**Figure 2:**
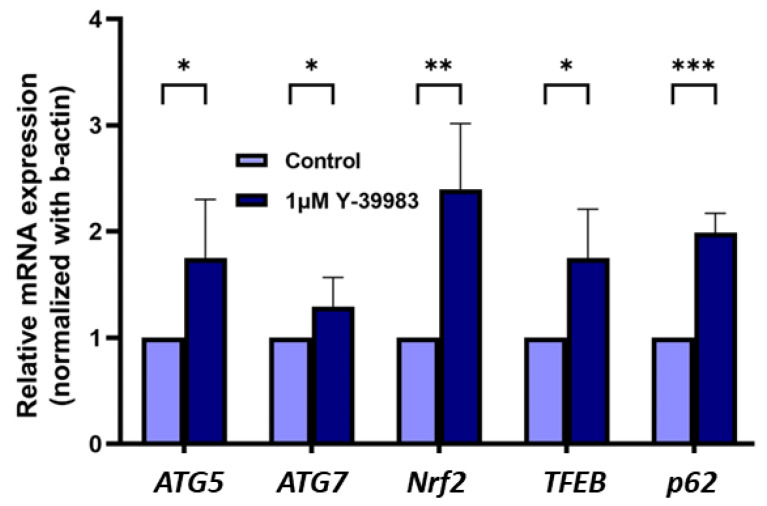
Effects of Y-39983 (1 μM) on the relative mRNA levels of autophagy genes *ATG5*, *ATG7*, *Nrf2*, *TFEB*, and *p62* in ARPE-19 cells following treatment with Y-39983 and the vehicle control (n = 5. *: *p* < 0.05, **: *p* < 0.01, and ***: *p* < 0.001; Student’s *t*-test).

Using reverse transcription followed by real-time quantitative PCR (qPCR), we showed that 1 µM Y-39983 significantly increased the mRNA levels of multiple autophagy genes, including *ATG5*, *ATG7*, *NRF2*, *TFEB*, and *p62*, in ARPE-19 cells after 48 h of treatment (Figure 2). Among the genes we investigated, *NRF2* and *p62* showed the most prominent increase in gene expression levels at 2.3-fold and 1.9-fold above the vehicle control, respectively.

**Figure 3:**
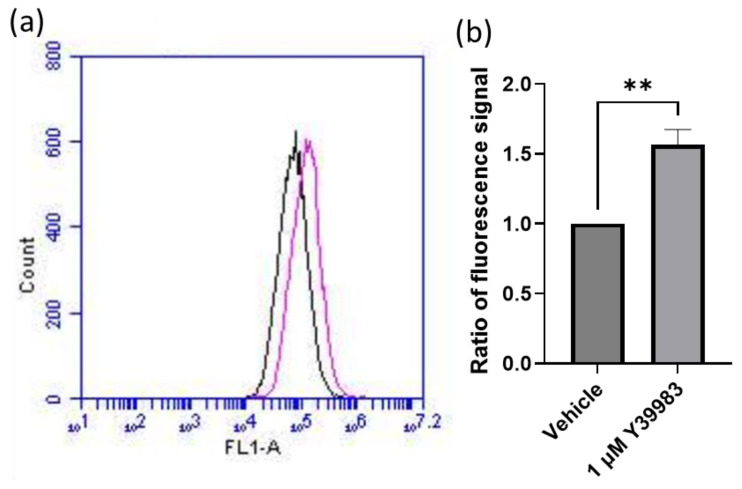
Y-39983 (1 μM) upregulated autophagic vacuole formation in ARPE-19 cells. ARPE-19 cells were incubated with Y-39983 (1 μM) or the vehicle for 48 h. Autophagic vacuoles in the cells were fluorescently labeled and quantified using a BD Accuri C6 Flow Cytometer. (**a**) A representative flow cytometry graph shows autophagic vacuoles in ARPE-19 cells treated with Y-39983 (pink line) or the vehicle (black line). (**b**) Y-39983 upregulated the autophagic vacuole formation by 56% at 1 μM when compared to the control. (Data represent the mean ± SEM; n = 5. **: *p* < 0.01; Student’s *t*-test).

We subsequently measured the autophagic vacuoles activity of ARPE-19 cells in the presence of the vehicle or Y-39983 (1 μM) using a fluorescent dye that selectively accumulates in autophagic vacuoles. Y-39983 upregulated autophagic vacuoles by 56% in ARPE-19 cells compared to the vehicle-treated control (Figure 3).

### 3.3. Protein Identification and Quantitation

**Figure 4:**
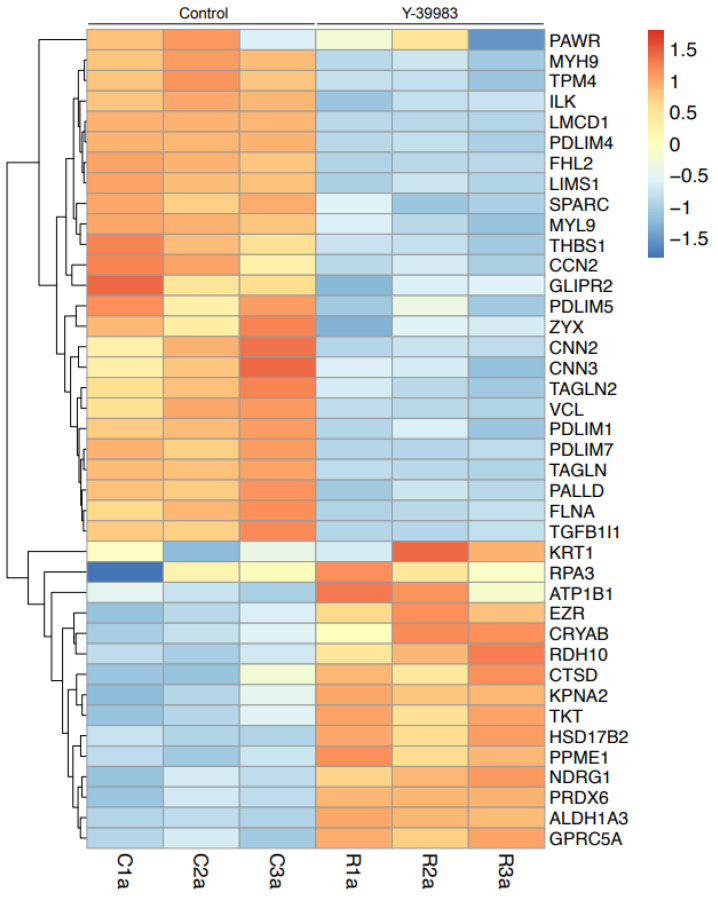
Heatmap of differentially expressed proteins (DEPs) in Y39983-treated vs. control samples. Each row corresponds to a unique gene coding the DEP, while each column represents an individual sample within the respective groups (technical replicates were averaged). Red and blue represent upregulation and downregulation, respectively. The scale bar indicates the intensity of the differential expression. Dendrogram on the left indicates hierarchical clustering (n = 3 for both treated and control groups).

Using Zeno SWATH data-independent analysis (DIA), a total of 54,146 proteins from all biological samples (n = 3 each group) and their two technical replicates were identified at the 1% False Discovery Rate (FDR). DEPs listed by the OneOmics cloud platform (SCIEX, Framingham, MA, USA) were filtered with the parameters as described in the Materials and Methods section to obtain reliable quantification. The analysis of samples treated with Y-39983 revealed 33 statistically significant DEPs, including 10 that were upregulated and 23 that were downregulated (Table 2 and Figure 4). Following the application of the cut-off values (fold change ≥ 1.3 or ≤ −1.3, *p*-value < 0.05) as described in the Materials and Methods section, THBS1 was among the proteins identified as a significantly downregulated protein.

### 3.4. Pathway Analysis of DEPs

All DEPs were imported into the iPathwayGuide commercial software (https://advaitabio.com/bioinformatics/ipathwayguide/, accessed on 9 August 2023) for a pathway analysis. The results indicated that four pathways were significantly regulated by Y-39983 treatment (adjusted *p*-value < 0.05, post-Bonferroni correction). The most significant pathway identified was “focal adhesion”, within which THBS1 was highlighted as a key regulated protein (Supplementary Figure S2).

### 3.5. Y-39983 Reduced THBS1 Gene and Protein Expression

**Figure 5:**
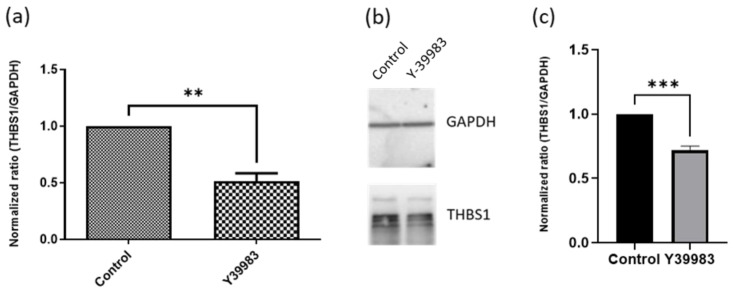
Expression of THBS1 mRNA and protein after Y-39983 treatment. (**a**) Effect of Y-39983 on the THBS1 mRNA levels in ARPE-19 cells as assessed by reverse transcriptase qPCR (n = 7; ** *p* < 0.01, Student’s *t*-test). (**b**) Representative Western blot of THBS1 in ARPE-19 cells. (**c**) Densitometry analysis of the THBS1 ratio (n = 3; ** *p* < 0.01, *** *p* < 0.001, Student’s *t*-test). The data represent the mean ± SEM.

Upon identifying THBS1 as one of the DEPs in the proteomic analysis, further experiments were performed to validate if THBS1 was truly downregulated in Y-39983-treated ARPE-19 cells. ARPE-19 cells were treated with 1 µM Y-39983 for 48 h. Western blot and qPCR analyses showed a reduction in THBS1 protein and mRNA expression, respectively (Figure 5). The results were consistent with the proteomic data, as the gene transcription level of *THBS1* in ARPE-19 cells was reduced by 48% after Y-39983 treatment (Figure 5a). Protein expression of THBS1 in Western blot analysis was significantly reduced by 28% (*p* < 0.001), as shown in Figure 5b,c.

### 3.6. Gene Silencing and Peptide Blocking of THBS1 Enhances Autophagy Activity

**Figure 6:**
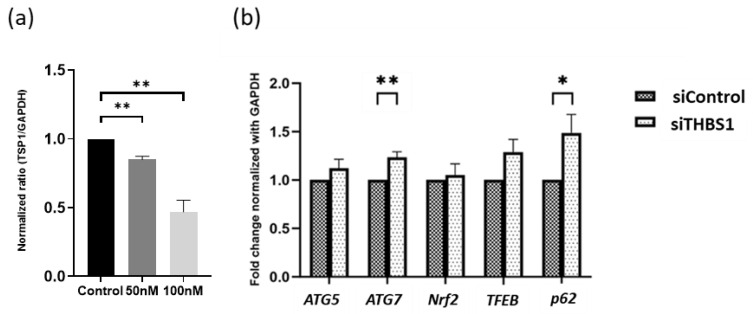
Effects of THBS1-siRNA knockdown on autophagy gene expression. (**a**) ARPE-19 cells were transfected with THBS1-siRNA or negative control siRNA for 24 h (n = 3 per group; ** *p* < 0.01, one-way ANOVA). (**b**) The mRNA levels of autophagy-related genes in ARPE-19 cells after siRNA treatment for 24 h (n = 5; * *p* < 0.05 and ** *p* < 0.01, Student’s *t*-test). The data represent the mean ± SEM.

**Figure 7:**
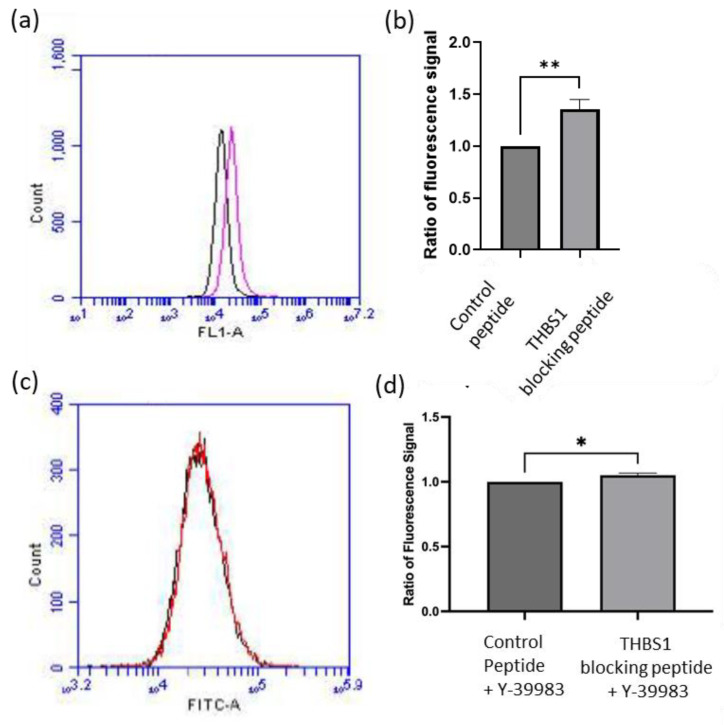
THBS1 blocking peptide upregulated autophagic vacuoles in ARPE-19 cells. (**a**) Representative flow cytometry graph showing autophagic vacuole quantification in ARPE-19 cells treated with THBS1 blocking peptide (pink) or control peptide (black), respectively. (**b**) THBS1 LSKL inhibitor peptide upregulated autophagic vacuoles by 35% at 5 μM when compared to the SLLK control peptide (n = 7, **: *p* < 0.01; Student’s *t*-test). (**c**) A representative flow cytometry graph shows autophagic vacuoles in ARPE-19 cells treated with Y-39983 with THBS1 blocking peptide (red line) or control peptide (black line). (**d**) Y-39983 upregulated autophagic vacuole formation by about 5% at 1 mM with LSKL peptide when compared to the control SLLK. (Data represent the mean ± SEM; n = 3; *: *p* < 0.05; Student’s *t*-test).

To investigate if THBS1 mediated the effects of Y-39983 on autophagy in APRE-19 cells, experiments using small interfering RNA (siRNA) against *THBS1* and a THBS1 blocking peptide were performed. A dose-dependent suppression of *THBS1* expression was observed after 24 h of anti-THBS1 siRNA treatment compared to the negative control siRNA (Figure 6a). The *THBS1* mRNA levels were significantly reduced by 53% after silencing with 100 pmol *THBS1* siRNA (*p* < 0.01). Thus, the concentration of 100 pmol *THBS1* siRNA was chosen for subsequent experiments. Figure 6b shows the transcription level of the autophagy genes after silencing *THBS1* for 24 h in ARPE-19 cells. The mRNA transcription level was numerically increased in all tested autophagy genes. However, the difference was statistically significant only for *ATG7* and *p62*, the two mRNAs with the most pronounced increase. Autophagic vacuoles were also measured in ARPE-19 cells treated with THBS1 blocking peptide. The blocking of THBS1 using 5 μM of the selective inhibitor LSKL peptide upregulated autophagic vacuole formation by 35% in ARPE-19 cells compared to the 5 μM control SLLK peptide (Figure 7a,b). After THBS1 blocking, Y-39983 could only increase autophagic vacuoles by approximately 5% in ARPE-19 cells (Figure 7c,d), indicating that Y-39983 induced autophagy through the regulation of THBS1 expression and function.

## 4. Discussion

In this study, the effects of Y-39983, a potent ROCK-inhibitor, on ARPE-19 cells were evaluated. It was demonstrated that Y-39983 induced changes in the morphology of ARPE-19 cells. Cell morphology is, in large part, determined by the cytoskeleton, which maintains cell shape by providing structural support to the cell [33]. Apart from this, the cytoskeleton also plays crucial roles in intracellular trafficking and signal transmission between extracellular and intracellular space [33]. Cytoskeletal arrangement can affect the polarity of RPE and thus influence RPE functioning and retinal homeostasis [33]. Consequently, changes in the cytoskeleton of ARPE-19 cells caused by treatment of Y-39983 not only altered the cell morphology but may also impact cellular functions, such as cell motility and cellular adhesion. Therefore, alteration in the cell morphology implies changes in cytoskeletal organization in Y-39983-treated ARPE-19 cells. Importantly, Y-39983 did not significantly affect cell viability, ruling out that any of the effects observed on autophagy genes and autophagic vacuoles were artifacts of reduced cell viability.

Apart from altering cell morphology, the measurement of autophagic vacuoles showed that Y-39983 upregulated autophagic vacuoles in ARPE-19 cells. Autophagy is a pivotal pathway for protecting RPE cells against oxidative stress and lipofuscin accumulation; the dysfunction of this pathway makes RPE cells more vulnerable to oxidative stress and increases the risk for AMD [8]. Our findings of an increased expression of autophagy genes and autophagic vacuoles in ARPE-19 cells after treatment with Y-39983 demonstrate that Y-39983 promotes autophagy in ARPE-19 cells. Previous studies showed that an increase in the p62 protein can indicate the inhibition of autophagy, followed by the reduced degradation of p62 and an increase in p62 protein abundance [34]. However, in this study, the *p62* mRNA levels were increased, which is not indicative of reduced protein degradation [35]. Of note, the upregulation of *p62* mRNA has also been observed in starvation-induced autophagy [36], consistent with the findings in this study. The finding of potent autophagy induction in human RPE cells by Y-39983 is of great interest, because it suggests that autophagy in the RPE can be pharmacologically upregulated with compounds that have been approved for the treatment of glaucoma.

In addition, the differential protein expression profile of ARPE-19 cells was investigated after Y-39983 treatment. Bioinformatic analysis revealed 33 statistically significant DEPs after ROCK inhibitor treatment. Of the 33 DEPs, 10 were upregulated, and 23 were downregulated. THBS1 was among the proteins identified as statistically significantly downregulated. The treatment of ARPE-19 cells with THBS1 siRNA or a THBS1 blocking peptide resulted in increased autophagic vacuole formation. The addition of Y-39983 to ARPE-19 already treated with THBS1 blocking peptide resulted in only a minimal change of autophagic vacuole formation. Hence, it is likely that Y-39983-mediated reduction of THBS1 protein abundance is the key mechanism underlying the autophagy-promoting effects of ROCK inhibition. Therefore, THBS1 may be a potential target for future AMD therapies. However, THBS1 is a secreted glycoprotein that plays a wide range of roles in cell-to-cell and cell-to-matrix interactions, and the reduction of THBS1 may also affect biological pathways other than autophagy. THBS1 is known to possess anti-angiogenic properties, most notably by inhibiting vascular endothelial growth factor (VEGF), a promoter of angiogenesis and a hallmark molecule for wet AMD [37,38]. Hence, a theoretical concern for the downregulation of THBS1 in RPE cells by ROCK inhibitors is the creation of a microenvironment that would promote neovascularization and wet AMD by preventing the inhibition of proangiogenic molecules such as VEGF. This concern is further heightened by previous studies, which have demonstrated that retinal endothelial cells from *THBS1* knockout mice had enhanced proangiogenic signaling properties and increased choroidal neovascularization [39,40]. Thus, while ROCK inhibition may protect RPE cells by enhancing autophagy, the downregulation of THBS1 could increase the risk for the development of wet AMD. Further studies dissecting the role of ROCK inhibitors in the autophagy and neovascularization pathways are warranted.

In this study, ARPE-19 cells were treated with 1 μM of Y-39983, a highly selective and potent ROCK inhibitor. The treatment did not significantly affect cell viability but caused a change in morphology—the formation of satellite-shaped cells, consistent with the known effects of ROCK on the cytoskeleton. In addition, Y-39983 increased the expression of autophagy genes and increased autophagic vacuole formation, demonstrating its influence in the autophagic process. Proteomic analysis, subsequent Western blotting, and reverse transcriptase qPCR assays confirmed that THBS1 was a major downregulated protein after Y-39983 treatment. Following mRNA knockdown and peptide blocking of THBS1, autophagic vacuole formation increased, and ROCK inhibition did not increase autophagic vacuole formation after THBS1 blockade, indicating that the autophagy effects exerted by ROCK inhibitors were primarily mediated by THBS1. THBS1 is a multifaceted protein with potential connections to autophagy and ROCK inhibition, which makes it an interesting target for therapeutic approaches in AMD and potentially other ocular conditions.

Despite these findings, our procedures only involved treating ARPE-19 cells with ROCK inhibitor only. Hydroquinone (HQ) or H_2_O_2_ exposure, such as those from smoking and other oxidative stress, are often chronic. Although our present study demonstrated that Y-39983 did not show the loss of cell viability, whether Y-39983 would offer a protective effect under chronic HQ or H_2_O_2_ exposure remains questionable. Therefore, further investigation on the effects of Y-39983 on HQ- or H_2_O_2_-treated cells may be required to evaluate its therapeutic potential in treating AMD.

## Figures and Tables

**Table 1:** Primer sequences for RT-qPCR.

Gene	Primer	Primer Sequence
*ATG5*	forward	5’-AAGCTGTTTCGTCCTGTGGC-3’
	reverse	5’-CCGGGTAGCTCAGATGTTCA-3’
*ATG7*	forward	5’-CGTTGCCCACAGCATCATCTTC-3’
	reverse	5’-TCCCATGCCTCCTTTCTGGTTC-3’
*Nrf2*	forward	5’-ACACGGTCCACAGCTCATC-3’
	reverse	5’-TGTCAATCAAATCCATGTCCTG-3’
*p62*	forward	5’-TGCCCAGACTACGACTTGTG-3’
	reverse	5’-AGTGTCCGTGTTTCACCTTCC-3’
*TFEB*	forward	5’-CCAGAAGCGAGAGCTCACAGAT-3’
	reverse	5’-TGTGATTGTCTTTCTTCTGCCG-3’
*THBS1*	forward	5’-CGTCCTGTTCCTGATGCATG-3’
	reverse	5’-CCAGGAGAGCTTCTTCCACA-3’
*GAPDH*	forward	5’-GATTTGGTCGTATTGGGCGC-3’
	reverse	5’-TGGACTCCACGACGTACTCA-3’

**Table 2:** Identification, fold change, and *p*-values of differentially expressed protein after Y39983 treatment. Protein expressions are depicted by color, with yellow indicating upregulated proteins and blue indicating downregulated proteins.

Protein Number	UniProt Accession Number	Protein Name	Gene ID	Fold Change	*p*-Value
1	Q8NFJ5	Retinoic acid-induced protein 3	GPRC5A	2.199	<0.001
2	P47895	Retinaldehyde dehydrogenase 3	ALDH1A3	2.196	<0.001
3	P37059	17-beta-hydroxysteroid dehydrogenase type 2	HSD17B2	1.922	<0.001
4	Q8IZV5	Retinol dehydrogenase 10	RDH10	1.852	0.010
5	Q92597	Protein NDRG1	NDRG1	1.612	<0.001
6	P30041	Peroxiredoxin-6	PRDX6	1.514	0.030
7	P02511	Alpha-crystallin B chain	CRYAB	1.386	<0.001
8	P35244	Replication protein A 14 kDa subunit	RPA3	1.341	<0.001
9	Q9Y570	Protein phosphatase methylesterase 1	PPME1	1.340	0.002
10	P15311	Ezrin	EZR	1.294	0.002
11	P21333	Filamin-A	FLNA	−1.294	<0.001
12	P37802	Transgelin-2	TAGLN2	−1.295	<0.001
13	P07996	Thrombospondin-1	THBS1	−1.345	0.009
14	Q96HC4	PDZ and LIM domain protein 5	PDLIM5	−1.360	0.008
15	P35579	Myosin-9	MYH9	−1.429	<0.001
16	O00151	PDZ and LIM domain protein 1	PDLIM1	−1.429	0.002
17	P48059	LIM and senescent cell antigen-like-containing domain protein 1	LIMS1	−1.467	<0.001
18	Q99439	Calponin-2	CNN2	−1.480	0.019
19	Q96IZ0	PRKC apoptosis WT1 regulator protein	PAWR	−1.494	<0.001
20	Q13418	Integrin-linked protein kinase	ILK	−1.506	<0.001
21	P09486	SPARC	SPARC	−1.525	<0.001
22	Q15942	Zyxin	ZYX	−1.681	<0.001
23	P29279	CCN family member 2	CCN2	−1.710	<0.001
24	Q8WX93	Palladin	PALLD	−1.763	<0.001
25	Q9NR12	PDZ and LIM domain protein 7	PDLIM7	−1.777	<0.001
26	P24844	Myosin regulatory light polypeptide 9	MYL9	−1.806	<0.001
27	P67936	Tropomyosin alpha-4 chain	TPM4	−1.817	<0.001
28	Q9NZU5	LIM and cysteine-rich domains protein 1	LMCD1	−2.082	<0.001
29	Q9H4G4	Golgi-associated plant pathogenesis-related protein 1	GLIPR2	−2.115	<0.001
30	Q01995	Transgelin	TAGLN	−2.209	<0.001
31	P50479	PDZ and LIM domain protein 4	PDLIM4	−2.237	<0.001
32	O43294	Transforming growth factor beta-1-induced transcript 1	TGFB1I1	−2.619	<0.001
33	Q14192	Four and a half LIM domains protein 2	FHL2	−2.892	<0.001

## Data Availability

Proteomic data are available via the ProteomeXchange Consortium with identifier PXD051479.

## Supplementary Materials

The following supporting information can be downloaded at https://www.mdpi.com/article/10.3390/cells13221907/s1: Figure S1: Cell viability of ARPE-19 cells incubated with control and different concentrations of Y-39983 (0.01 µM, 0.1 µM, 1 µM). Data were shown as mean ± SEM (N = 9, One-way ANOVA test); Figure S2: The 4 significant pathways associated with significantly regulated proteins were enriched by iPathwayGuide. Notably, THBS1 was a regulated protein within the most significant pathway—focal adhesion. The criteria for pathway significance were set at an adjusted *p*-value of less than 0.05, determined by Fisher’s exact test, and subsequently corrected using the Bonferroni method.
