# Supplementary material for: Thrombospondin 1 Mediates Autophagy Upon Inhibition of the Rho-Associated Protein Kinase Inhibitor
Source: Cells. 2024 Nov 18;13(22):1907. doi: 10.3390/cells13221907 (PMC11593289; doi:10.3390/cells13221907)
Supplement: Supplementary file 1 [file cells-13-01907-s001.zip › cells-3257687-supplementary.pdf]

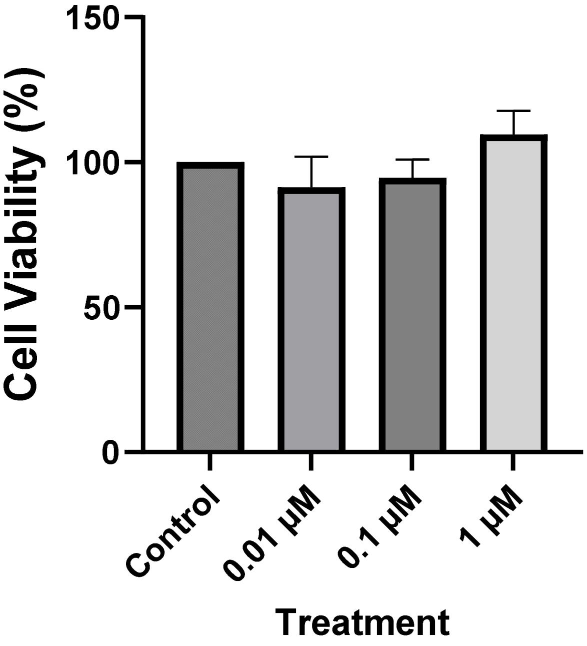

Figure S1. Cell viability of ARPE-19 cells incubated with control and different concentrations of Y-39983 (0.01 μM, 0.1 μM, 1 μM). Data were shown as mean ± SEM (N = 9, One-way ANOVA test).

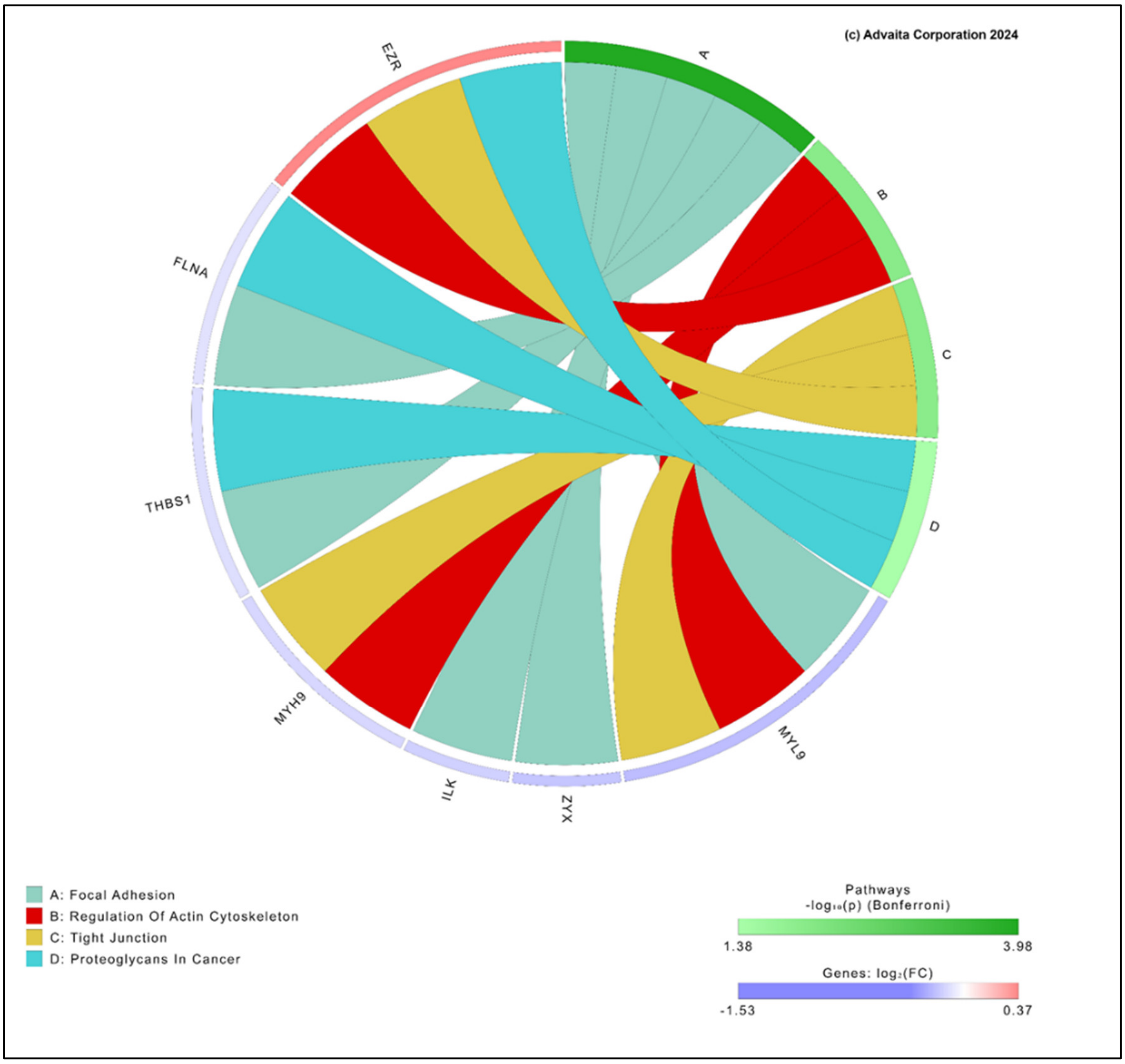

Figure S2. The 4 significant pathways associated with significantly regulated proteins were enriched by iPathwayGuide. Notably, THBS1 was a regulated protein within the most significant pathway—focal adhesion. The criteria for pathway significance were set at an adjusted *p*-value of less than 0.05, determined by Fisher's exact test, and subsequently corrected using the Bonferroni method.
